# Ovariotomy and Parotitis

**Published:** 1881-08

**Authors:** 


### Ovariotomy and Parotitis.

It is an established fact that orchitis and inflammation of the parotid may mutually complicate each other. Moreover, there has been observed a relation between inflammation of the salivary gland in question and that of the external genital organs and the ovaries. Facts in support of this opinion are found in the works of Bouteiller, Meynet, Peter, and Billroth. Schroeder, who had never met parotitis as a complication of operations on the female genital organs, has just seen it as a sequel of five ovariotomies, two of which proved fatal (*Il Morgagni*). He comes to the conclusion that parotitis is a grave complication of gynæcological operations.—*Medical Press and Circular.*

